# Relationship between monocyte/lymphocyte ratio and non-culprit plaque vulnerability in patients with acute coronary syndrome: An optical coherence tomography study

**DOI:** 10.1097/MD.0000000000021562

**Published:** 2020-10-09

**Authors:** Ting-yu Zhang, Qi Zhao, Ze-sen Liu, Chao-yi Zhang, Jie Yang, Kang Meng

**Affiliations:** a Department of Cardiology, Beijing Anzhen Hospital, Capital Medical University; b Department of Cardiology, Beijing Luhe Hospital, Capital Medical University; c Department of Cardiology, Beijing Zhongguanchun Hospital, Beijing, China.

**Keywords:** monocyte-to-lymphocyte ratio, optical coherence tomography, plaque vulnerability

## Abstract

The importance of monocyte/lymphocyte ratio (MLR) has been indicated in the initiation and progression of coronary artery disease. However, few previous researches demonstrated the relationship between MLR and plaque vulnerability. We aimed to investigate coronary non-culprit plaque vulnerability in patients with acute coronary syndrome (ACS) by optical coherence tomography (OCT).

A total of 72 ACS patients who underwent coronary angiography and OCT test in Beijing Anzhen Hospital were included in this retrospective study. The plaque vulnerability and plaque morphology were assessed by OCT.

The non-culprit plaque in high MLR group exhibited more vulnerable features, characterizing as thinner thickness of fibrous cap (*P* = .013), greater maximum lipid core angle (*P* = .010) and longer lipid plaque length (*P* = .041). A prominently negative liner relation was found between MLR and thickness of fibrous cap (R = –0.225, *P* = .005). Meanwhile, the proportion of OCT-detected thin cap fibro-atheroma (TCFA) (*P* = .014) and plaque rupture (*P* = .017) were higher in high MLR group. Most importantly, multivariable logistic regression analysis showed MLR level was identified as an independent contributor to the presence of TCFA (OR:3.316, 95%: 1.448–7.593, *P* = .005). MLR could differentiate TCFA with a sensitivity of 60.0% and a specificity of 85.1%.

Circulating MLR level has potential value in identifying the presence of vulnerable plaque in patients with ACS. MLR, as a non- invasive biomarker of inflammation, may be valuable in revealing plaque vulnerability.

## 1 Introduction

As the most severe form of coronary artery disease (CAD), acute coronary syndrome (ACS) is the leading cause of mortality in recent decades. Development of a thrombus on the background of an atherosclerotic plaque rupture, which subsequently lead to arterial occlusion, is the major mechanism responsible for the majority of ACS. In recent years, it has been widely realized that it is plaque composition rather than plaque size or stenosis severity significant for plaque rupture and subsequent thrombosis.^[1]^ Vulnerable plaques were described as plaques with thin fibrous caps and large lipid core.

Inflammation reaction plays a significant role in the occurrence and progress of atherosclerosis.^[2,3]^ Previous researches have demonstrated that increased level of monocytes, neutrophils and decreased level lymphocytes have closely association with the cardiovascular adverse outcomes.^[4,5]^ Recently, Hanhua et al reported that monocyte-to-lymphocyte ratio (MLR) was an independent risk factor of the presence of CAD, and significantly associated with coronary lesion severity.^[6,7]^ Furthermore, Chen et al reported that MLR was an independent predictor for major adverse cardiovascular event in patients with non-ST segment elevated myocardial infarction.^[7]^ It was also demonstrated that increased MLR level was associated with higher morality in patients with acute heart failure.^[8]^ However, the relationship between the MLR level and plaque vulnerability has not been sufficiently evaluated. On account of high resolution (10 μm), optical coherence tomography (OCT) can accurately identify the features of coronary atherosclerotic plaques and observe microstructure of coronary arteries such as fibrous cap, lipid nucleus and intravascular thrombosis of plaques. Meanwhile, OCT has advantages in differentiating the thin cap fibro-atheroma (TCFA).^[9,10]^ In this regard, our present study aimed to identify the relationship between MLR level and plaque vulnerability evaluated by OCT in patients with ACS.

## 2 Methods

### 2.1 Study population

From May 2015 to May 2017, a total of 72 patients with ACS who underwent coronary angiography and OCT test in one of the cardiovascular centers in Beijing Anzhen Hospital in this retrospective study. This research was approved by the Ethics committee of Beijing Anzhen Hospital affiliated to the Capital Medical University, and all patients signed the informed consent form. We collected general data, laboratory examination, and relevant treatment data of all patients. The definition of ACS included acute myocardial infarction (AMI) and unstable angina pectoris. AMI was diagnosed as acute myocardial injury and clinical evidence of acute myocardial ischemia, meanwhile cardiac troponin levels rose and/or fell at least once exceed the 99th percentile of the upper limit of normal. St-segment elevated myocardial infarction was defined as acute myocardial infarction with new st-segment elevation ≥1 mm in at least of 2 adjacent leads except V2 and V3, while non-ST segment elevated myocardial infarction was defined as no st-segment elevation at the time of myocardial infarction. We defined unstable angina pectoris as primary angina, aggravating exertional angina, or angina at rest episodes with ischemia changes on electrocardiogram. Patients with ACS were not in acute stages (within 2 weeks) in the study. We defined culprit lesion as the lesion with the most severe stenosis or with evidence of recent plaque disruption based on angiographic findings. In the present study, we included non-culprit plaques as the research objects.

The exclusion criteria included AMI in acute phase, cardiogenic shock, left ventricular ejection fraction < 40%, renal insufficiency, malignant, autoimmune disease, hematologic disease, active systemic inflammatory, or in medication, such as steroid.

### 2.2 Laboratory analysis

For all patients, venous blood samples were tested by the laboratory of Beijing Anzhen Hospital. The blood samples were collected after the acute phase of acute myocardial infarction. The MLR value was measured as the ratio of monocyte counts to lymphocyte counts.

### 2.3 Coronary angiography and analysis

Coronary angiography is performed through radial or femoral artery pathways. All patients received a loading dose of aspirin (300 mg) and clopidogrel (300 mg) at least 6 hours before angiography. The images were analysis by 2 cardiac doctors.

### 2.4 OCT analysis

OCT imaging was obtained using the commercially available, frequency-domain C7-XR OCT Intravascular Imaging System (St Jude Medical, St Paul, MN) and Dragonfly catheter (Light Lab Imaging, Inc) before the intervention. OCT images and measurements were analyzed using light Lab Imaging by 2 independent cardiac doctors. The indistinct OCT image and length of vessel images less 50 mm was excluded.

**Figure 1:**
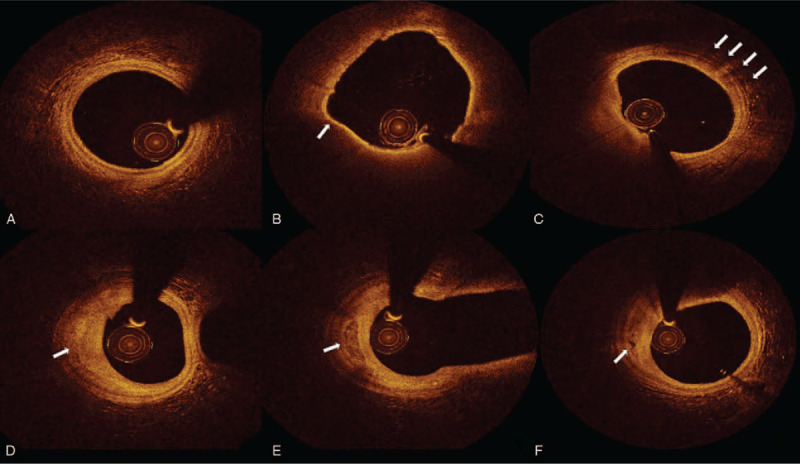
Coronary plaques of OCT images. A. Normal vascular structure B. Thin cap fibroatheroma and arrow showed the thickness of fibrosis cap was 60 μm C. Macrophages infiltration D. Fibrosis plaque E. Calcium plaque F. Microchannel.

OCT was used to identify the presence of fibrous plaques, lipid plaques, calcified plaques, macrophage infiltration, microchannels, cholesterol crystals, plaque rupture, and thrombus, as well as the imaging characteristics of lipid plaques.^[11–15]^ Normal vascular structure as shown in Figure 1A. Lipid plaque was defined as a diffusely bordered, signal-poor region with an overlying signal-rich bands. Each plaque was at least 5 mm apart from each other or at least 5 mm away from the scaffold edge. When the lipid plaque was identified, the lipid length was measured with the system's own measuring tool, and the lipid core arc of the lipid plaque was measured every 1 mm. At the thinnest place of the lipid plaque fibrous cap, measured 3 times the thickness of the fibrous cap and calculated the average as the plaque thickness of fibrous cap (FCT). The length of the lipid plaque, maximum lipid core angle (max lipid arc), average lipid core angle (mean lipid arc) and lipid index (defined as average lipid arc times lipid plaque length) were recorded respectively. Thin cap fibro-atheroma (TCFA) (as shown in Fig. 1B) was defined as a lipid plaque whose thickness of fibrous was less than 65 μm and whose lipid core was larger than 2 quadrants. Macrophages was defined as dots or bands with high reflection and strong attenuation, and they often form radial shadows after dots of high signal (as shown in Fig. 1C). Fibrous plaque was defined as a signal-rich region with scattering (as shown in Fig. 1D). Calcified plaques were defined as low-signal or non-uniform areas with sharp edges (as shown in Fig. 1E). The micro channel was defined as a 50 to 300 μm, low-signal and sharp edge hollow-like structure, which needs to be observed in at least 3 continuous sections (as shown in Fig. 1F). Cholesterol crystals was defined as thin linear regions with high signal intensity and low attenuation. Plaque rupture was defined as the disruption of plaque fibrous cap continuity with cavity formation. Intracoronary thrombus was defined as a mass (diameter 250 μm) attached to the luminal surface or floating within the lumen.

### 2.5 Statistical analysis

All statistical analyses were performed by SPSS 21.0. Measurement data in accordance with normal distribution was mean ± standard deviation, the comparison between groups using t-test. Non-normal distribution of the measurement data using the median (25th –75th percentiles), the comparison between the group using Mann–Whitney *U* test. Counting data using example (%), the comparison between groups is X^2^ test or Fisher exact test. Receiver operating characteristic (ROC) curve analysis was performed to examine the diagnostic value of MLR in predicting the OCT-detected TCFA. Liner regression analysis was used to assess the correlations between MLR and each plaque component. Multivariate logistic regression analysis was carried out to identify the independent risk factors of the TCFA. Variables that had a clinically possible relation with TCFA or appeared to be significant in univariate model were introduced into multivariable analysis. Both were bilateral tests and *P* < .05 were statistically significant.

## 3 Results

We finally enrolled 72 patients with 120 plaques in this study. Based on the median of MLR value: 0.21, subjects were classified into 2 groups: low MLR group (MLR ≤ 0.21, 36 subjects with 61 plaques) and high MLR group (MLR > 0.21, 36 subjects with 59 plaques).

### 3.1 Baseline clinical characteristics

The baseline demographic and clinical characteristics between the 2 groups were shown in Tables 1 and 2. The patients in high MLR group showed higher ages (*P* = .005) and lower BMI values (*P* = .035). Compared with the low MLR group, those with high MLR had higher CRP (*P* = .023), monocyte count (*P* < .001), and lower lymphocyte count (*P* < .001). There was no statistical significance in other index between 2 groups.

**Table 1:**
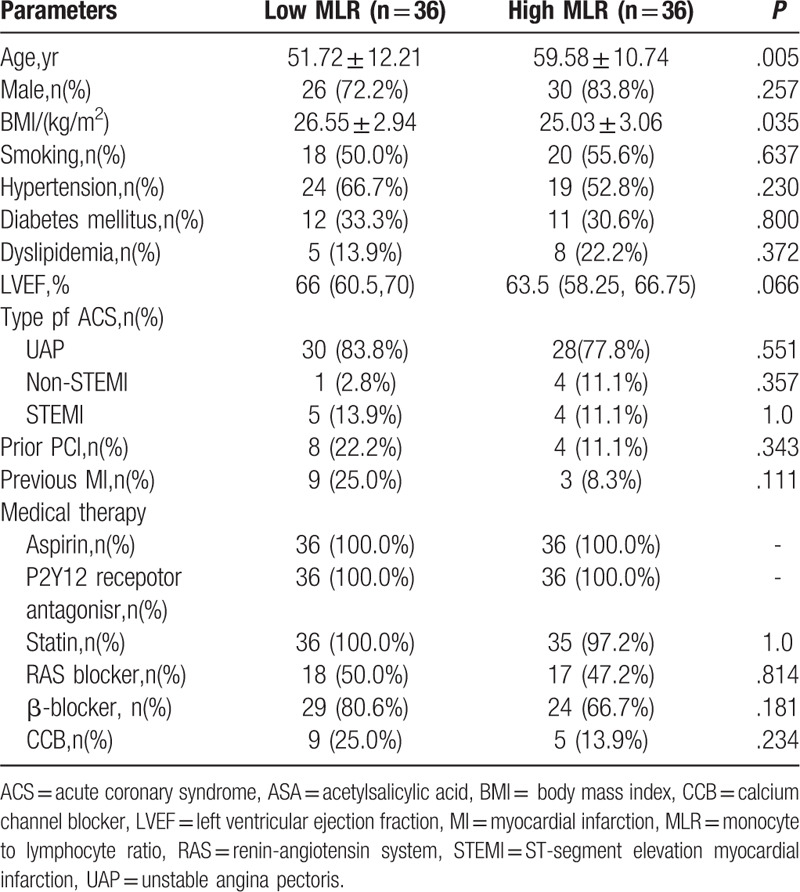
Baseline characteristics.

**Table 2:**
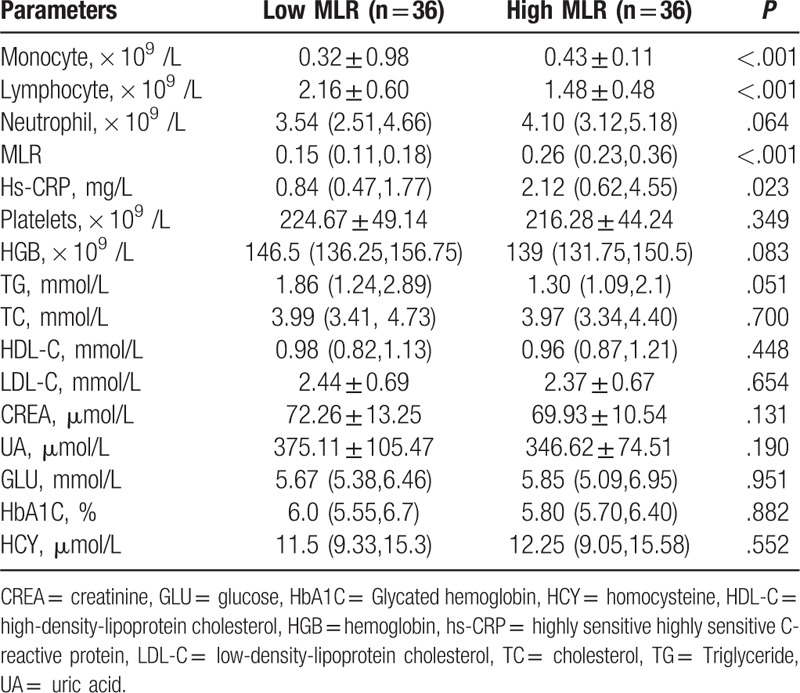
Laboratory parameters.

### 3.2 Coronary angiography and OCT image findings

No prominent difference was found in the number and characteristics of lesions between the high and low MLR group, as shown in Table 3.

**Table 3:**
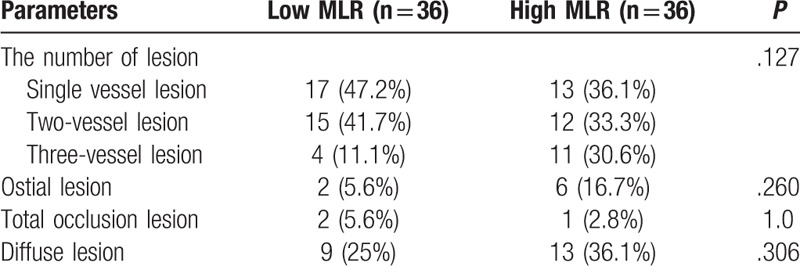
Angiographic findings.

**Figure 2:**
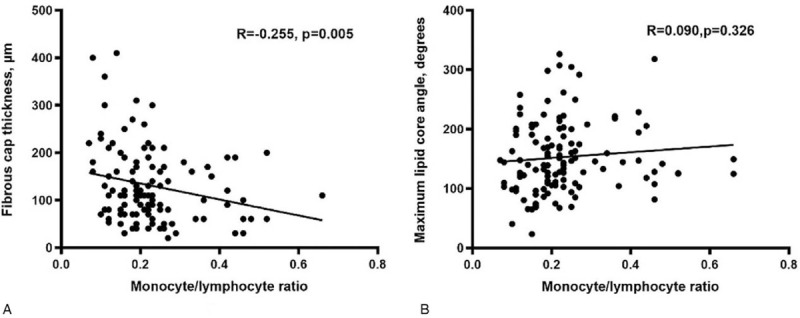
Correlation analysis of monocyte/lymphocyte ratio (MLR) and relative plaque components of the non-culprit plaque assessed by optical coherence tomography. (A) Fibrous cap thickness, (B) Maximum lipid core angle.

OCT images showed that the ratio of TCFA (47.2% vs 19.4%, *P* = .014) and plaque rupture (38.9% vs 13.9%) were higher in the high MLR group. Meanwhile, the high MLR group showed thinner fibrous cap thickness (FCT)(112.37 ± 60.24 vs 153.49 ± 73.29 μm, P = 0.013), greater maximum lipid angle (167.36 ± 62.33 vs 138.79 ± 56.37°, *P* = .010) and longer lipid plaque length (6.34 ± 3.12 vs 4.50 ± 2.21 mm,*P* = .041) than the low MLR group, as shown in Table 4. Furthermore, a prominently negative liner relation was found between MLR and FCT (R = –0.225, *P* = .005) (Fig. 2A).

**Table 4:**
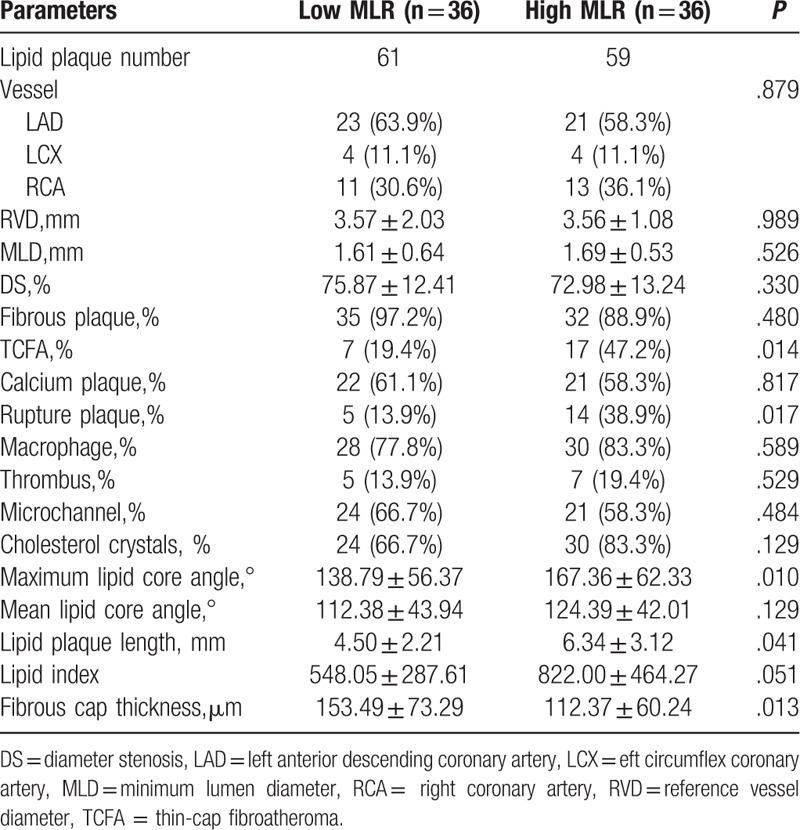
Coronary plaques of optical coherence tomography images.

### 3.3 Risk factors of TCFA

**Figure 3:**
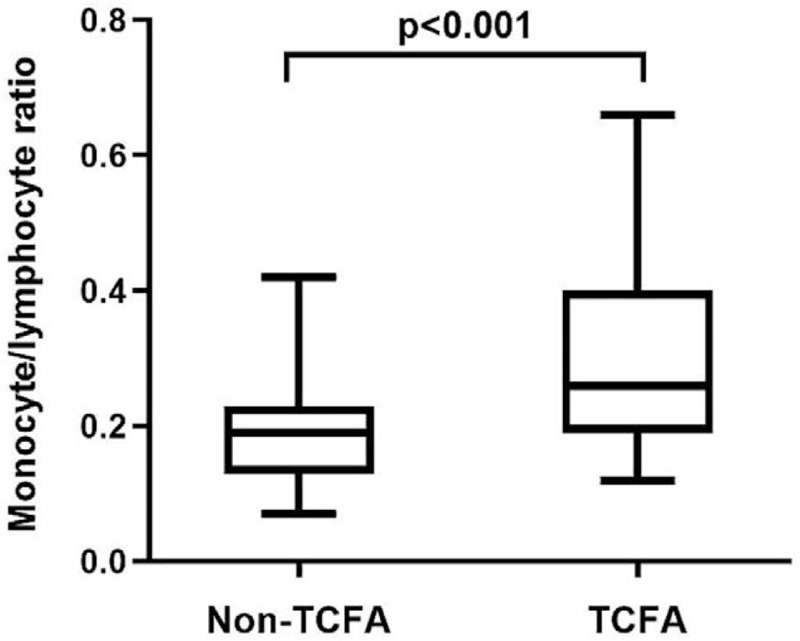
Comparison of MLR levels between patients with and without TCFA.

According to the presence of OCT-detected TCFA, patients were classified into TCFA group (25 patients) and non-TCFA group (47 patients). Patients with TCFA had significantly higher MLR level compared with those without TCFA (*P* < .001) (Fig. 3). Multivariate logistic regression analysis showed that MLR was the independent risk factor of TCFA, after adjusting for confounding factors (OR: 3.316, 95% CI: 1.448–7.593, *P* = .005), as shown in Table 5.

**Table 5:**
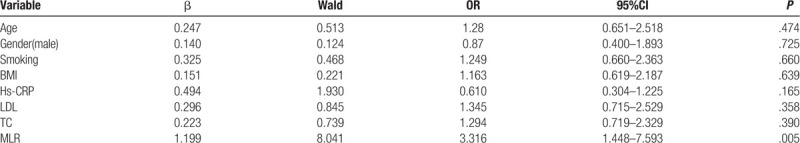
Logistic regression analysis of = thin cap fibro-atheroma.

**Figure 4:**
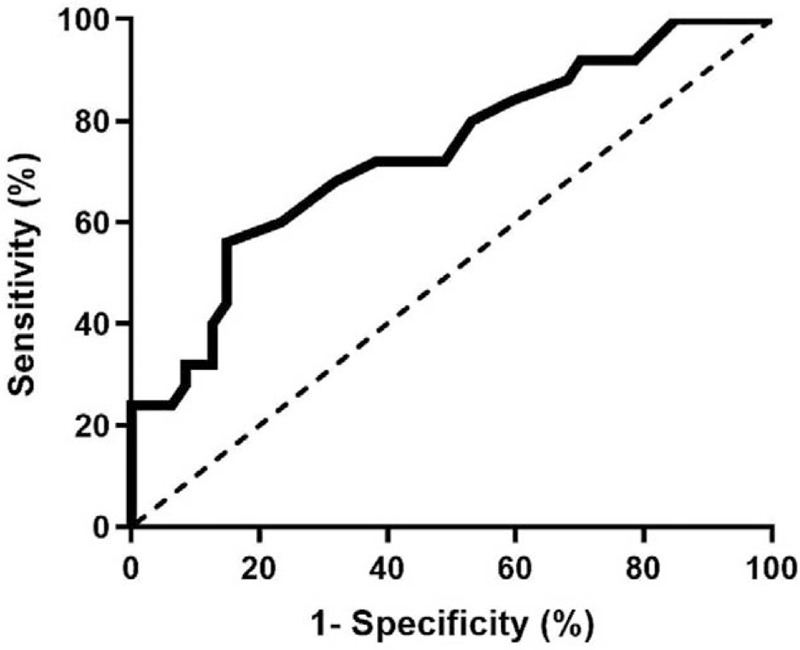
ROC curve analysis evaluating predictive value of MLR.

The predictive value of MLR for TCFA was further examined by ROC curve analysis. ROC curve analysis indicated that the area under the curve was 0.741(95% CI 0.619–0.864, *P* = .001), which showed a good predictive accuracy of MLR for the presence of TCFA in patients with ACS (Fig. 4). The baseline MLR level at 0.25 was identified as the optimal cutoff point to predict the risk of TCFA with a sensitivity of 60% and a specificity of 85.1%.

## 4 Discussion

In recent years, many advances have been made in the identification and prediction of coronary plaque stability using non-invasive biomarkers. Our trial was the first to use the OCT test to evaluate the relationship between MLR levels and non-culprit coronary plaque vulnerability in ACS patients. In the present study of ACS patients with high MLR, non-culprit plaque presented more vulnerable features, characterizing as thinner FCT, greater maximum lipid angle and longer lipid plaque length. Meanwhile, the incidence of plaque rupture and OCT-detected TCFA were higher in high MLR group. And high MLR level was demonstrated to be an independent risk factor of TCFA.

MLR is the ratio of monocytes to lymphocytes and the combined indicator of the 2 inflammatory factors. As the major mechanism of atherosclerosis, inflammation response has a great impact on the occurrence and development of atherosclerosis lesions, and eventually result in plaque instability and severely adverse outcomes related to CAD.^[16,17]^ One general feature of atherosclerotic lesions is the presence of inflammatory cells, such as macrophages, T cells. Monocytes and lymphocytes in the leukocyte subtype are crucial cells in the inflammatory process. Previous studies have shown that monocytes are closely related to many vital steps in cardiovascular pathology and atherosclerosis. Macrophages, derived from the circulating monocytes, influence the initiation and progression of atherosclerotic plaque by participating foam cells formation and inducing the production of inflammatory cytokines, matrix metalloproteinases, and reactive oxidative species.^[18–20]^ A novel therapeutic target associated with monocytes phenotype modulation has been used for cardiovascular diseases prevention and treatment.^[21]^ Low lymphocyte count has been found to be a critical factor in atherosclerotic progress where inflammation responses plays a vital role. A decrease in lymphocyte cells was related to worse clinical outcomes in patients with cardiovascular disease, especially ACS. Furthermore, it has been demonstrated that increased lymphocytes apoptosis has negative impact on ejection fraction, area of myocardial necrosis and microvascular function in patients with ACS, especially acute myocardial infarction (AMI).^[22–24]^ As a combination of two different biomarkers of inflammation, MLR can provide additive value to the evaluation of cardiovascular diseases. It could be concluded that high monocytes, low lymphocytes or both are associated with higher risk of CAD. Meanwhile, MLR is a widely available, inexpensive, and non-invasive biomarker.

Coronary unstable plaques are more prone to plaque rupture with subsequent thrombogenesis leading to episodes of acute coronary syndrome. Lipid-rich plaques with thin fibrous caps are the highest-risk and most vulnerable plaque phenotype. The TCFA, which characterizes as thin fibrous cap and a great lipid angle, tends to cause major adverse cardiovascular events.^[25]^ OCT, as a high-resolution imaging modality, has great advantages in identifying TCFA and providing detailed information about the lipid plaque, such as FCT. Meanwhile, OCT enables in vivo visualization of plaque burden, plaque morphology and response to therapy. In the present study, the plaque in high MLR group showed more vulnerable characteristics which had more prone to plaque rupture, leading to the occurrence of adverse cardiac events. And a conspicuously negative relation was found between MLR and FCT. The measurement of the necrotic lipid core and the FCT play crucial roles in the dynamics of plaque destabilization. The pathophysiological mechanisms between elevated MLR and plaque vulnerability may be related to immune-inflammatory response.^[1–3]^ Inflammation, proteolysis, and decreased collagen fiber content are likely to lead to plaque rupture.

The presence of vulnerable plaques is the main cause of ACS.^[26]^ Previous study demonstrated that MLR level was independently with TCFA in the patients with stable angina. The vulnerable plaque was identified by intravascular ultrasound, which cannot provide specific morphology characteristics of plaque, such as the thickness of plaque components.^[27]^ Compared with the stable angina patients, ACS patients showed more intravascular ultrasound-derived TCFA in non-culprit lesions. In this study, the advantage of OCT in identifying vulnerable plaques was utilized to provide a direct link between MLR and the vulnerability of coronary atherosclerotic plaques in ACS patients, such as FCT and lipid core angle. The present study found the incidence of TCFA and plaque rupture were higher in high MLR group. After adjusting for potential confounding factors, MLR was an independent predictable factor of TCFA. Based on ROC curve analysis, the cut-off value for MLR level was 0.25, with a sensitivity of 60.0% and specificity of 85.1% for predicting TCFA. Compared with monocytes count or lymphocytes count, we considered that MLR, as a combination of 2 inflammatory indicators, may has a greater advantage in identifying plaque vulnerability in ACS patients.

### 4.1 Study limitations

The current study has several potential limitations. Firstly, this study was a small sample and single-center retrospective study, this was a major limitation and source of bias for the findings. More trials are needed to confirm this result. Secondly, we were concerned with non-culprit atherosclerotic plaques in culprit vessels, but not non-target lesions. Thirdly, the study was a cross-sectional trial, but no follow-up data and prognostic implications were analyzed. Despite these limitations, this study firstly explored the value of MLR level in vulnerability to coronary plaque in ACS patients.

## 5 Conclusion

In conclusion, the coronary atherosclerotic plaques in high MLR group exhibited more vulnerable characteristics. Meanwhile, elevated MLR level was independently with OCT-detected TCFA in culprit vessels in patients with ACS. These results reflected that circulating MLR, as a noninvasive and widely available inflammation biomarker, may has a potential value for determining atherosclerotic plaque vulnerability in the setting of ACS.

## Author contributions

**Conceived and designed the study:** Tingyu Zhang and Qi Zhao.

**Data and images collection and analyzed:** Zensen Liu and Chaoyi Zhang.

**Quality control the study and revision:** Kang Meng.

**Wrote the manuscript:** Tingyu Zhang, Qi Zhao and Jie Yang.

All authors read and approved the final manuscript.

## Abbreviations

Abbreviations: ACS = acute coronary syndrome, AMI = acute myocardial infarction, CAD = coronary artery disease, FCT = thickness of fibrous cap, MLR = monocyte-to-lymphocyte ratio, OCT = optical coherence tomography, ROC = receiver operating characteristic, TCFA = thin cap fibro-atheroma.
